# A Role for Early Cardiac Function in Cardiac Morphogenesis

**DOI:** 10.1371/journal.pbio.0020138

**Published:** 2004-05-11

## Abstract

xx

The heart starts beating and pumps blood through the body long before it has achieved its mature architecture. In theory, this provides a chance for cardiac function to sculpt cardiac structure, an intriguing possibility for developmental biologists, and one of potentially great clinical import for cardiologists seeking to identify the causes of (often fatal) cardiac anomalies. In this issue of *PLoS Biology,* Thomas Bartman et al. use the powerful tools afforded by zebrafish genetics to dissect the early steps of heart valve formation. In the process, they provide evidence for a causal relationship between the early function of the heart and its final structure.[Fig pbio-0020138-g001]

**Figure pbio-0020138-g001:**
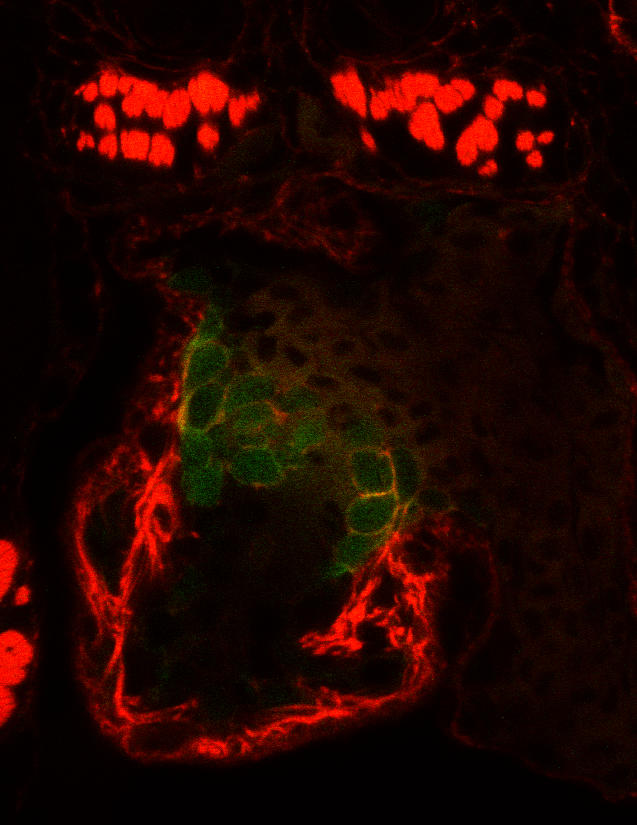
tie2::GFP+ cells form the endocardial cushions

At the time of its first beat, the vertebrate heart is little more than a tube, lined on its outside by a myocardial cell layer whose contractions (the heartbeats) power blood flow, and on its inside by an endocardial cell layer, an extension of the inner wall of the connecting blood vessels. What it lacks still are valves and septae, the fibrous gates that subdivide the mature heart into atrial and ventricular chambers, and control the directionality of blood flow. These structures derive from the endocardium in a process that begins—shortly after the establishment of blood flow—with the local accumulation of endocardial cells into what are known as endocardial cushions (ECs).

The zebrafish lends itself well to large-scale genetic screens, and powerful genomic tools are now available to efficiently identify the gene affected by any mutation. The authors have used genetic screens to identify several mutations that affect early cardiac function or morphology. Heart anomalies are easy to detect in zebrafish, and can be examined in real time and in live specimens because the embryos develop outside the mother and are fully transparent. Using a fluorescent molecular marker highly expressed in the ECs, the authors narrowed in on mutations that result in valve defects, and identified a mutant they named *cardiofunk (cfk),* which was devoid of ECs. Genetic mapping of the *cfk* mutation revealed a single sequence change in a gene encoding a novel actin molecule that is most closely related to the sarcomeric actins found in sarcomeres, the contracting organelles of muscle cells. The result was surprising because contractions are not a property of endocardial cells. Using RNA detection assays, the authors show that the cfk gene is in fact expressed in the myocardium, rather than in the endocardium. It therefore appears that the inability to form ECs in cfk mutants does not reside in the endocardium per se, but is an indirect consequence of a myocardial anomaly.

The *cfk* mutation introduces a single amino acid change in the actin protein, and through detailed biochemical analyses, the authors show that the mutant actin is impaired in its ability to assemble into fibers in vitro. What might be the consequence in vivo? The authors note that *cfk* mutants display abnormal heart contractions prior to the development of their EC defect. Support for the notion that myocardial contractions are required for EC formation comes from the examination of *silent-heart (sih)* mutants. *sih* mutants, which lack a heatbeat, have been shown to harbor a mutation in troponin T, one of the motors of actin contractions; the authors find that *sih* mutants also fail to develop ECs. The mechanisms linking myocardial contractions and cushion formation remain unclear. Blood flow may be a trigger, though the authors find that ECs can develop even in the presence of pharmacological compounds that abolish it. The characterization of additional mutants should help answer this question.

Valve or septal defects represent 40% of cardiac anomalies in humans. Bartman and colleagues suggest that, by analogy with zebrafish, some may result from congenital defects affecting very early myocardial function. Their work thus opens new avenues for the early detection of human cardiac malfunctions and malformations.

